# Modeling Parkinson’s Disease in *Drosophila*: What Have We Learned for Dominant Traits?

**DOI:** 10.3389/fneur.2018.00228

**Published:** 2018-04-09

**Authors:** Yulan Xiong, Jianzhong Yu

**Affiliations:** Department of Anatomy and Physiology, Kansas State University College of Veterinary Medicine, Manhattan, KS, United States

**Keywords:** Parkinson’s disease, *Drosophila*, modeling, leucine-rich repeat kinase 2, α-synuclein, glucocerebrosidase, vacuolar protein sorting 35

## Abstract

Parkinson’s disease (PD) is recognized as the second most common neurodegenerative disorder after Alzheimer’s disease. Unfortunately, there is no cure or proven disease modifying therapy for PD. The recent discovery of a number of genes involved in both sporadic and familial forms of PD has enabled disease modeling in easily manipulable model systems. Various model systems have been developed to study the pathobiology of PD and provided tremendous insights into the molecular mechanisms underlying dopaminergic neurodegeneration. Among all the model systems, the power of *Drosophila* has revealed many genetic factors involved in the various pathways, and provided potential therapeutic targets. This review focuses on *Drosophila* models of PD, with emphasis on how *Drosophila* models have provided new insights into the mutations of dominant genes causing PD and what are the convergent mechanisms.

## Introduction

Parkinson’s disease (PD) is recognized as the most common movement disorder and the second most common neurodegenerative disorder after Alzheimer’s disease (1). The classical motor features including akinesia, resting tremor, muscle rigidity, and postural imbalance are clinical symptoms in PD patients, and the none-motor features including cognitive impairment, psychiatric symptoms, sleep disorders, autonomic dysfunction, pain, and fatigue also frequently occur (1). The progressive degeneration of dopaminergic (DA) neurons in the substantia nigra pars compacta is the cause for the cardinal symptoms (2). Although the majority of PD cases appear to be sporadic, the identification of causative genes that cause familial forms of PD has led to important insights into the pathogenesis of this progressive neurodegenerative disease (3). To date, genes encoding α-synuclein (α-Syn), leucine-rich repeat kinase 2 (LRRK2), Parkin, phosphatase and tensin homolog deleted on chromosome 10-induced putative kinase 1 (PINK1), DJ-1, vacuolar protein sorting 35 (VPS35), and glucocerebrosidase (GBA), among others are associated with genetic forms of PD that closely resemble idiopathic PD (3–8). Among these genes, *LRRK2*, α*-synuclein, GBA*, and *VPS35* are the dominant traits, and *parkin, DJ-1*, and *PINK1* are the recessive genes. Various model systems have been developed to study the function of PD-causing genes and provided tremendous insights into the molecular mechanisms underlying DA neurodegeneration. While few of genetic models in rodents recapitulate the cardinal features of PD, the power of *Drosophila* has revealed many genetic factors involved in the various pathways, and provided potential therapeutic targets. Here, we focus on the dominant genes causing PD. We discuss how *Drosophila* models have provided new insights into the mutations of dominant genes causing PD and what are the convergent mechanisms.

## *Drosophila* as a Model in the Study of PD

*Drosophila melanogaster*, as a non-mammalian animal, provides a simple, yet powerful, *in vivo* system to model PD pathobiology. *Drosophila* has well-defined nerve system. Particularly, in adult brain, *Drosophila* has distinct DA neuronal clusters including about 200 DA neurons and displays complicated behaviors mimicking some human behaviors which are DA dependent. Both transgenic and knockout approaches have been utilized to develop *Drosophila* models of PD. The *Drosophila* Gal4/UAS system is a powerful tool for targeted transgene expression and has been used to direct selective expression of mutant PD genes. As a simple organism, *Drosophila* provides great advantages in conducting genome-wide modifier screenings and high-throughput drug screenings. Modifier screenings allow analyses of genome-wide genetic interactions based on the modification of given phenotypes and further identify components of diverse signaling pathways involved in PD pathogenesis.

Several steps can be taken to establish and utilize *Drosophila* models to study PD:

(1) Develop *Drosophila* lines carrying mutant PD genes,
(2) Characterize the phenotypes of the *Drosophila* models and determine whether they recapitulate the pathogenesis of the disease,
(3) Explore the molecular mechanisms underlying the phenotypes,
(4) Identify genetic modifiers that suppress or enhance the disease phenotypes through genetic screenings, and dissect the signaling pathways and pathogenic mechanisms involved in pathogenesis,
(5) Screen for drug candidates,
(6) Study the impact of environmental (e.g., toxin) or aging influence in combination with genetic factors on the pathogenesis of PD.

## Modeling LRRK2-Associated PD in *Drosophila*

Mutations in the *LRRK2* gene (PARK8, dardarin) is the most common known genetic cause of PD and cause late-onset, autosomal dominant PD with age-related penetrance and clinical features identical to late-onset sporadic PD (9, 10). LRRK2 is a large multifunctional protein about 280 kD. It includes two important enzymatic domains, which are a GTPase domain and a kinase domain, and several protein interaction domains including a LRRK2-specific repeat domain, a leucine-rich repeat, and a WD40 repeat (11–13). Disease causing mutations are found in both enzymatic domains, indicating their importance in disease pathogenesis (11, 12). The most prevalent LRRK2 mutation, G2019S, within the kinase domain, accounts for ~1% of sporadic late-onset PD and 5–6% of familial PD worldwide (14). In North African Arabs and Ashkenazi Jews, the frequency of LRRK2–G2019S mutation can be as high as 30–40% in PD patients (15, 16). Patients with the G2019S mutation exhibit Lewy bodies (LBs) in most cases and incomplete penetrance even at advanced ages (1). However, mutations in the GTPase domain such as R1441 C/G often vary on LB pathology and exhibit nearly complete penetrance (9, 10). This suggests that these pathogenic mutations may cause disease *via* distinct pathogenic pathways/mechanisms. Tremendous work suggests LRRK2 GTPase and kinase enzyme activities may reciprocally regulate each other to direct LRRK2 functions in diverse cellular signaling pathways (17, 18). LRRK2 is demonstrated to be involved in protein translation, vesicle trafficking, mitochondrial function, lysosomal–autophagy, and cytoskeletal dynamics (13, 18–22). However, how LRRK2 mutations cause neurodegeneration in PD still need to be defined. To this end, various animal models of LRRK2-associated PD have been generated (23–25). Among these models, LRRK2 *Drosophila* models have provided unique and critical insights on LRRK2 functions (Table 1).

**Table 1:** *Drosophila* models for leucine-rich repeat kinase 2 (LRRK2)-associated Parkinson’s disease.

Genetic manipulations	References	LRRK2 variants	Neurodegeneration	Motor activities/life span	Other functions
Knockout	(26)	dLRRK	Tyrosine hydroxylase (TH) neurons: no changes TH neurons shrunken TH staining ↓	Locomotor activity ↓	ND
	(27)	dLRRK	No changes	Life span ↓	Sensitive to hydrogen peroxide, not to paraquat, rotenone, and β-mercaptoethanol
	(28)	dLRRK	TH neurons: no changes; DA content ↑	Life span ↓ Fertility ↓ Malformed abdomen	Hydrogen peroxide ↓ Paraquat ↓
	(29)	dLRRK	ND	Locomotor activity ↓	ND
Transgenic	(26)	dLRRK	No changes	No changes	ND
	(30)	hLRRK2	TH neurons ↓ No response to l-DOPA Retinal degeneration	Locomotor activity ↓ Life span ↓ Response to l-DOPA	ND
		hG2019S	TH neurons ↓↓ No response to l-DOPA Retinal degeneration	Locomotor activity ↓ Life span ↓ Response to l-DOPA	ND
	(28)	dLRRK	No changes	ND	No changes
		dY1383C dI1915T	TH neurons: no changes TH staining ↓ DA content ↓	ND	Hydrogen peroxide ↑ Paraquat ↑
	(31)	hLRRK2 (at 29°C)	TH neurons ↓ Retinal degeneration	Locomotor activity: 10 days ↓ 20 days ↑ Life span ↑ Fertility ↑	Rotenone ↑
		hI1122V (at 29°C) hY1699C (at 29°C) hI2020T (at 29°C)	TH neurons ↓ the most with I2020T Retinal degeneration	Locomotor activity: 10 days ↓ 20 days ↑ Life span ↑ in hY1699C, hI2020T Fertility ↑ in hI1122V, hI2020T	Rotenone ↑
	(32)	hLRRK2	No changes	No changes	No changes
		hG2019S hY1699C hG2385R	TH neurons ↓	Locomotor activity ↓ Life span ↓	hG2019S, hG2385R ↑ hY1699C no change
	(33)	hLRRK2	ND	Locomotor activity: no changes Life span ↓	Dendritic ends ↓
		hG2019S	TH neurons ↓	Locomotor activity ↓↓ Life span ↓↓	Dendritic ends ↓↓ Axon degeneration ↑
		hR1441C	ND	Locomotor activity ↓	Dendritic ends ↓
		hG2385R		Life span ↓	
	(34)	hLRRK2	ND	ND	Visual function: no changes
		hG2019S	ND	ND	Visual function ↓
		hI1122V hR1441C hY1383C hI1915T hI2020T hG2385R hG2019/K 1906M	ND	ND	Visual function: no changes
	(35)	hLRRK2 hG2019S	ND	Locomotor activity: no changes	Axon transport: no changes
		hR1441C hY1699C	ND	Locomotor activity ↓	Axon transport ↓
		dR1069C dY1383C	ND	Locomotor activity ↓	Axon transport ↓
	(36)	hG2019S hI2020T	ND	Bradykinesia, akinesia, hypokinesia, and increased tremor	Proboscis extension response ↓
		hR1441C hG2019/K 1906M	ND	No changes	No changes

*ND, not determined; O/E, overexpression; ↑, increased; ↓, decreased*.

### LRRK2 *Drosophila* Models

#### LRRK2 Knockout *Drosophila* Models

*Drosophila* has a single human LRRK2 homolog, dLRRK. Residues changed by PD-causing mutations in human LRRK2 are highly conserved in *dLRRK* (Table 1). To study the physiological function of endogenous LRRK2, *dLRRK* loss-of-function mutant fly lines have been generated. One major line used in the studies is LRRK^e03680^ from the Exelixis Collection at the Harvard Medical School. It was generated with piggyBac element insertion in the intron between exon 5 and exon 6 of *dLRRK* gene. In characterization of dLRRK knockout *Drosophila* model on PD related pathogenesis, one study reported knockout of dLRRK exhibited a decrease in tyrosine hydroxylase (TH) immunostaining, shrunken DA neurons, and locomotor activity deficits (26), while three studies reported that the homozygous mutant flies developed unchanged number and pattern of DA neurons as well as a normal life span (27–29). Furthermore, the sensitivity of those dLRRK2 knockout flies response to oxidative stress have been examined. Wang et al. showed that *dLRRK* mutant flies are selectively sensitive to H_2_O_2_ (27). By contrast, a report by Imai et al. demonstrated that *dLRRK* knockout flies are relatively resistant to paraquat and H_2_O_2_ treatment (28). Thus, the exact role of dLRRK in PD-related pathogenesis remains elusive. As the majority of studies supported that *dLRRK* is not required for DA neurons survival and this is consistent with the results from LRRK2 knockout rodents (mice or rats), the general agreement is that LRRK2-induced neuronal toxicity is from a gain-of-function but not a loss-of-function mechanism.

#### LRRK2 Transgenic *Drosophila* Models

Patients carrying heterozygou s or homozygous LRRK2 pathogenic mutations have similar disease risk and progression, supporting LRRK2 dominant nature (1) (Table 1). Indeed, in contrast to *dLRRK* loss-of-function mutant, overexpression of either human LRRK2 (hLRRK2) or dLRRK pathogenic mutations in flies leads to an age-dependent DA neuronal loss and DA-responsive locomotor deficits (28, 30–33, 36, 37). Notably, different LRRK2 mutations cause different phenotypes of the degeneration. One study demonstrated that dopaminergic expression of LRRK2 G2019S led to non-autonomous neurodegeneration in visual system (34). This degeneration is specific to G2019S mutation and dependent on kinase activity. Another report showed that GTPase-COR domain mutations R1441C or Y1699C, but not G2019S, preferentially inhibits axonal transport in *Drosophila* and causes locomotor deficits (35). This suggests that the defects depend on LRRK2 GTPase activity (35). Recently, Cording et al. reported that expressing either the G2019S or I2020T but not R1441C, or kinase dead LRRK2 in DA neurons reduces proboscis extension response, with bradykinesia, akinesia, and tremor (36). These studies support the possibility that different LRRK2 pathogenic mutations act at distinct pathways and cause disease *via* distinct pathogenic mechanisms. The LRRK2 transgenic fly models support the gain-of-function of LRRK2 in PD pathogenesis.

### LRRK2 *Drosophila* Models Reveal LRRK2 Functions in PD

#### LRRK2 Functions in Vesicular Trafficking

Vesicular trafficking has been implicated to play crucial roles in neurodegeneration (38). LRRK2 *Drosophila* models have provided extensive evidence of potential roles for LRRK2 in various vesicle trafficking processes including endocytosis, ER-Golgi and retromer trafficking, and autophagy–lysosomal pathways (39). dLRRK was reported to localize to the membranes of late endosomes and lysosomes, physically and functionally interacts with Rab7, a key mediator of late endosomal transport and lysosome biogenesis (40). Nonsense alleles in dLRRK induced by ethyl methanesulfonate causes striking defects in the autophagy–lysosomal pathways (41). Furthermore, LRRK2 has been shown to interact with clathrin-light chains to limit Rac1 activation on endosomes (42). Importantly, studies in *Drosophila* show that LRRK2 phosphorylates endophilin A (EndoA), a central component of synaptic endocytosis, and synaptojanin 1 (SJ1), a synaptic vesicle protein which was recently linked to recessive PD (37, 43–45). LRRK2 regulates EndoA and SJ1 by phosphorylation at synapses, which facilitates synaptic endocytosis through clathrin uncoating at the synaptic terminals. In addition, LRRK2’s role in retromer and ER–Golgi trafficking was highlighted by genetic interactions between LRRK2 and VPS35, Rab7L1, ArfGAP1 in *Drosophila* (46–49). Moreover, dLRRK has been demonstrated to regulate axonal transport and Golgi outpost dynamics in dendrites through the golgin Lava lamp (35, 50). Taken together, these studies strongly support the roles of LRRK2 in vesicular trafficking processes, which may provide potential mechanisms for α-Syn accumulation in LRRK2-associated PD.

#### LRRK2 Functions in Protein Translation Machinery

The first evidence of LRRK2 function in protein translation was demonstrated in *Drosophila* (28). In this study, both dLRRK and human LRRK2 can phosphorylate eukaryotic initiation factor 4E-binding protein (4E-BP), a negative regulator of eukaryotic initiation factor 4E-mediated protein translation and a key mediator downstream of mTOR signaling to various stress responses (28). The notion that LRRK2 functions in protein translation was further strengthened by the study from the same group that LRRK2 interacts with the microRNA pathway to regulate protein synthesis (51). However, these studies were done in *Drosophila* system and need to be extended to mammalian systems. Subsequently, Martin and colleagues, using LRRK2 *Drosophila* model and human DA neurons, demonstrated that LRRK2 phosphorylates ribosome protein s15 to regulate protein translation and mediate LRRK2-induced neurodegeneration (52). Recently, Penney et al. showed that LRRK2 targets Furin1 to promote cap-dependent translation, which is required for LRRK2 synaptic function (53). Taken together, these findings support convergent evidence that LRRK2 regulates protein translation machinery directly or indirectly, which could be a potential therapeutic avenue for LRRK2-associated PD.

#### LRRK2 Functions in Dendritic Degeneration and Synaptic Dysfunction

Mutant LRRK2 functions in dendritic degeneration were first revealed by the evidence that LRRK2 G2019S induces mislocalization of the axonal protein tau in dendrites and in turn causes dendrite degeneration (33). This may act through tau phosphorylation by the glycogen synthase kinase 3β, which is promoted by LRRK2 G2019S (33). In addition, LRRK2 regulates synaptic morphology through phosphorylation of Futsch at the presynaptic compartments and interaction with 4E-BP at the postsynaptic site of the *Drosophila* neuromuscular junctions (NMJs) (54). Furthermore, a recent finding indicates that loss of dLRRK disrupts the retrograde synaptic compensation while overexpression of either dLRRK or hLRRK2 can induce a retrograde enhancement of presynaptic release (53). This regulation of synaptic homeostasis might act through a mechanism that LRRK2 promotes cap-dependent translation (53). These studies suggest that LRRK2 might regulate synaptic function in neural circuits.

### LRRK2 *Drosophila* Models as Platforms to Identify Potential Therapeutic Compounds

The genetic LRRK2 *Drosophila* models provide promising *in vivo* platforms for inhibitor identification and validation, and drug development. It has shown that sorafenib, curcumin, or GW5074 significantly suppressed LRRK2 PD-like phenotypes in *Drosophila* (55, 56). Melatonin attenuates hLRRK2-induced synaptic dysfunction and sleep disorders (57). Although candidate compounds have been used in these studies, they open the possibility of performing compound screens. Recently, Lin et al. identified compounds from the FDA-approved licensed drug library that could rescue LRRK2-induced neurite degeneration, motor disability, and DA neuron loss (58). Of 640 compounds, lovastatin had the highest lipophilicity, which facilitates crossing the blood–brain barrier (58). These studies provide significant steps toward the development of new drugs for treatment of LRRK2-associated PD.

## Modeling α-Syn-Associated PD in *Drosophila*

The discovery of the first missense mutation A53T in the *SNCA* gene in 1997 (59) and the insoluble aggregated α-Syn forms as the major component of LBs, a pathological hallmark of PD (60), heralded a new era in PD research. Since then, more *SNCA* pathogenic mutations as well as multiplications of *SNCA* have been identified as genetic causes of PD [review in Ref (61).]. In addition, multiple genome-wide association studies have identified *SNCA* as a risk factor for sporadic PD (62, 63). These findings revealed a central role of *SNCA* in PD. *SNCA* encodes α-Syn protein, a small protein with 140 amino acid residues. It is highly soluble and enriched at presynaptic terminals, where it binds lipids and regulates synaptic vesicle release and it has a propensity of self-aggregate to form oligomeric species and LB-like fibrils (64, 65). Multiple evidence suggest that oligomeric species of α-Syn, which are precursors for higher-order fibrillar aggregates in LBs, are pathogenically toxic and the culprits for neuronal degeneration (66). Recently, prion-like behavior of α-Syn has attracted a lot of attention and been debated in playing an important role in the pathogenesis of PD (67, 68). Although *Drosophila* have no homolog of *SNCA*, pathogenic mutations and multiplication of *SNCA* causing PD with dominant inheritance pattern implicates a toxic gain-of-function mechanism, which led to suitable transgenic modeling in fly by overexpressing wild-type or mutant α-Syn (69) (Table 2).

**Table 2:** Transgenic *Drosophila* models of α-Syn-associated Parkinson’s disease.

Transgenic systems	References	α-Syn variants	Neurodegeneration	Motor/non-motor activities and life span	Cellular functions
	(70)	α-Syn-WT A30P A53T	Tyrosine hydroxylase (TH) neurons ↓ Retinal degeneration ↑	Locomotor activity ↓	Filamentous intraneuronal inclusions containing α-Syn

	(71)	α-Syn-WT A30P A53T	TH neurons ↓	ND	Lewy body- and LN-like inclusions, Hsp70 protected against α-Syn–induced dopaminergic neuronal degeneration

	(72)	α-Syn-WT	ND	ND	Phosphorylation of α-Syn at S129 ↑
		A30P			Phosphorylation of α-Syn at S129 ↑↑
		A53T			Phosphorylation of α-Syn at S129 ↑↑↑

	(73)	α-Syn-WT A30P A53T	TH neurons: no changes No retinal degeneration	Locomotor activity: no changes	ND

	(74)	α-Syn-W	TH neurons ↓ Retinal degeneration ↑	ND	ND
		S129A	TH neuron: no changes No retinal degeneration		
		S129D	TH neurons ↓↓ Retinal degeneration ↑↑		

Gal4/UAS system	(75)	α-Syn-WT	TH neurons ↓	ND	α-Syn aggregation ↑
		α-SynΔ71–82aa	TH neurons: no changes		No α-Syn aggregation
		Syn 1–120aa	TH neurons ↓↓		α-Syn aggregation ↑↑

	(76)	α-Syn-WT	TH neurons: no changes	Locomotor activity: no changes	Soluble oligomers of α-Syn *in vitro*

		A30P A53T A56P	TH neurons ↓	Locomotor activity ↓	Soluble oligomers of α-Syn *in vitro* ↑

		A30P/A56P/A76P (TP)	TH neurons ↓↓	Locomotor activity ↓↓	Soluble oligomers of α-Syn *in vitro* ↑↑

	(77)	α-Syn-WT	TH neurons ↓ Retinal degeneration ↑	ND	Soluble oligomers of α-Syn ↑
		Y125F/Y133F/Y136 F (YF)	TH neurons ↓↓ Retinal degeneration ↑↑		Soluble oligomers of α-Syn ↑↑
		S129D	TH neurons ↓↓ Retinal degeneration ↑↑		Soluble oligomers of α-Syn ↑↑

	(78)	α-Syn-WT	ND	Sleep behavior normalCircadian locomotor activity defects ↑	ND
		A53T		Sleep behavior abnormal ↑ Circadian locomotor activity defects ↑	

		A30P/A56P/A76P (TP)	ND	Sleep behavior abnormal ↑↑ Circadian locomotor activity defects ↑↑	ND

	(79)	A30P	ND	Olfactory deficits	ND

	(80)	A30P	TH neurons ↓	Locomotor activity ↓ Anxiety ↑	ND

Q system	(81)	α-Syn-WT	TH neurons ↓↓	Locomotor activity ↓↓	α-Syn aggregation ↑↑

*ND, not determined; ↑, increased; ↓, decreased*.

### α-Syn Transgenic *Drosophila* Models

Feany and Bender first developed α-Syn transgenic *Drosophila* models by overexpressing either wild-type or familial mutants A53T and A30P of human α-Syn using the conventional Gal4/USA expression system (70). These models recapitulate the essential features of PD: adult-onset loss of DA neurons, filamentous intraneuronal inclusions containing α-Syn and locomotor dysfunction (70). In an independent study, Auluck et al. confirmed the phenotypes reported by Feany and Bender (71). In addition to these phenotypes, Chen et al. demonstrated olfactory deficits and elevated anxiety in a α-Syn transgenic *Drosophila* model expressing A30P (79, 80). There are deficits in two out of three olfactory modalities, odor discrimination and tested-olfactory acuity. A30P expression in dopamine neurons is necessary for both acuity and discrimination deficits. Gajula Balija et al. showed the non-motor symptoms such as an abnormal sleep-like behavior, locomotor deficits, and abnormal circadian periodicity when targeted expression of pre-fibrillar α-Syn mutants in a subset of serotonergic and DA neurons (78). In 2017, the Feany group expended the scope of their previous α-Syn transgenic *Drosophila* models using a binary expression system, the Q system, which relies on the transcriptional activation by the *Neurospora* protein QF2 to activate transgene expression (81). This new α-Syn *Drosophila* model shows robust neurodegeneration, early-onset locomotor deficits, and abundant α-Syn aggregation (81). Although there is some discrepancy over the strength of the phenotypes (73, 82), the α-Syn transgenic *Drosophila* models are widely used to delineate underlying pathogenic mechanisms and identify novel proteins mediating α-Syn toxicity.

### α-Syn *Drosophila* Models Reveal α-Syn Functions in PD

#### α-Syn Aggregation and Misfolding in α-Syn-Induced Neurotoxicity

Accumulating evidence revealed α-Syn aggregation and misfolding plays a central role in the pathogenesis of PD and synucleinopathies. Wild type or mutant α-Syn has been demonstrated to be aggregated as inclusions when overexpressing in flies (70, 71, 81). Structurally engineered α-Syn mutants with an increased oligomerization propensity increase neurotoxicity in *Drosophila* (76). Truncation of α-Syn contributes to aggregation and LB formation. Expression of α-Syn with a deletion of NAC domain (α-Syn Δ71–82) did not show evidence of α-Syn aggregation and any DA neurodegeneration, suggesting the essential role of NAC domain of α-Syn in aggregation and toxicity (75). By contrast, a C-terminal truncated α-Syn has ability to promote aggregation and enhance neurotoxicity (75). Interestingly, calpain-cleaved α-Syn fragments with similar molecular weight to truncated α-Syn have been identified in the PD/DLB patient brains and α-Syn-expressing flies (83). This suggests the physiological and pathological importance of the truncated α-Syn.

Protein quality control systems including molecular chaperones and protein degradation function as a defense mechanism against protein misfolding and aggregation. Identification of suppressors in these systems further supports a critical role of toxic oligomers and aggregation in α-Syn-induced neurotoxicity. Histone deacetylase 6 suppresses α-Syn-induced DA neurodegeneration and promotes the formation of α-Syn inclusions by reducing α-Syn oligomers (84). Auluck et al. demonstrated that increasing the level of chaperone Hsp70 ameliorated the toxicity of α-Syn to DA neurons while a reduction in chaperone activity enhanced α-Syn-induced DA neuronal loss in *Drosophila* system (71). In addition, decreased level of the mitochondrial chaperone protein tumor necrosis factor receptor associated protein-1 enhanced A53T-α-Syn-induced age-dependent DA neuron loss in fly (85). Molecular chaperones assist proper protein folding and thus protect against α-Syn misfolding and aggregation. If proteins have been misfolded and aggregated, they are cleared by degradation. The ubiquitin-protease system and the autophagy-lysosome systems are two major protein degradation systems. Using *Drosophila* and cell culture systems, Lee et al. demonstrated that co-expression of ubiquitin can rescue α-Syn-induced neurotoxicity. This neuroprotection is dependent on the formation of lysine 48 polyubiquitin linkage, which is known to target protein degradation (86). This observation is further strengthened by evidence that overexpression of the ubiquitin ligase Nedd4 can rescue α-Syn-induced degenerative phenotype in fly (87). Furthermore, the deubiquitinase Usp8 interacted and partly colocalized with α-Syn, and deubiquitinated K63-linked chains on α-Syn. Knockdown of Usp8 in fly reduced α-Syn levels and α-Syn-induced toxicity (88). In addition, Cullen et al. showed that the defect of cathepsin D, a major lysosomal aspartyl protease, enhanced α-Syn-induced neurodegeneration *in vivo* in *Drosophila* (89). Taken together, these results confirmed that protein quality control systems function as a protection mechanism against α-Syn aggregation and misfolding.

#### α-Syn Phosphorylation Controls Neurotoxicity Inclusion Formation

Phosphorylation of α-Syn plays a key role in the PD pathogenesis. Phosphorylation at Ser129 is the one extensively phosphorylated in brain tissues from PD patients and related disorders, suggesting a role for Ser129 phosphorylation in disease pathogenesis (90, 91). In transgenic flies, it has been demonstrated that human α-Syn is phosphorylated at Ser129, and phosphorylation increases with age as DA neurons degenerate, mimicking the pathogenic phenomena in PD patient (72). Later on, Chen and Feany generated transgenic flies carrying the mutations at S129 of α-Syn (S129A to block phosphorylation and S129D to mimic phosphorylation) (74). Using these transgenic lines, they demonstrated phosphorylation of S129 is critical for α-Syn-induced DA neuron degeneration, and blocking S129 phosphorylation increases inclusion formation (74). As increased number of inclusion bodies correlates with reduced toxicity, this study suggested inclusion bodies might have protective function. Recently, the Feany group reported that tyrosine and serine phosphorylation of α-Syn have opposing effects: levels of soluble oligomeric species of α-Syn were increased by serine 129 phosphorylation and decreased by tyrosine 125 phosphorylation, suggesting detrimental effects of S129 phosphorylation and a neuroprotective action of T125 phosphorylation (77). These studies reveal that phosphorylation of α-Syn plays an important role in α-Syn-induced inclusion body formation and DA neurodegeneration.

#### α-Syn Functions in Vesicular Trafficking

The first evidence of α-Syn functions in trafficking in animal models has been reported by Cooper et al. using a combination of a genetic screening in yeast and validation in α-Syn transgenic *Drosophila* models (92). In this study, Rab1 rescues the neuron loss in the flies (92). Recently, using *Drosophila* models of α-Syn toxicity, several reports have implicated α-Syn functions in vesicular trafficking particularly through the small GTPase Rab proteins. The endosomal recycling factor Rab11 was demonstrated to modulate synaptic vesicle size, decrease α-Syn aggregation and ameliorate several α-Syn-dependent phenotypes (93). Rab7, regulating trafficking of late endosomes and autophagosomes, and Rab8, modulating post-Golgi vesicle trafficking, rescue the locomotor deficit in α-Syn flies (94, 95). Notably, other PD genes such as LRRK2 and PINK1 have also recently been linked to Rab proteins (48, 96–98). Thus, determination of the precise mechanisms of Rabs-mediated functions in PD pathogenesis is warranted.

#### α-Syn Functions in Mitochondrial Dysfunction and Oxidative Stress

Oxidative stress and mitochondrial dysfunction have been proposed as important causative factors for the progression of PD. Botella et al. found that DA neurons are specifically sensitive to an environmental oxidative insult (hyperoxia) induced oxidative stress. The mutant forms of α-Syn enhanced the toxicity under this stress in the *Drosophila* model (99). In addition, the co-expression of Cu/Zn superoxide dismutase protects against mutant α-Syn-induced DA neuronal loss (99). The same group also demonstrated that dopamine, which produces reactive oxygen species, might be involved in the α-Syn-induced neurotoxicity through oxidative stress (100). Furthermore, α-Syn-induced neuronal death in *Drosophila* is enhanced by the mutants that promote glutathione synthesis and conjugation (101). Natural antioxidants attenuate locomotor deficits of α-Syn transgenic flies (102). GPI, an enzyme in glucose metabolism, acts as neuroprotection from α-Syn proteotoxicity in flies (103). Recently, Feany group provided evidence that the interaction of α-Syn with spectrin initiates pathological alteration of the actin cytoskeleton and downstream neurotoxicity, and consequent mitochondrial dysfunction through altered Drp1 localization (81). These results suggest that oxidative stress and mitochondrial dysfunction are features of α-Syn toxicity.

### α-Syn *Drosophila* Models as Platforms to Identify Potential Therapeutic Compounds

Based on the functions and mechanisms revealed by α-Syn *Drosophila* models, several pharmacological interventions have been developed in *Drosophila* to ameliorate α-Syn toxicity. Geldamamycin, an Hsp90 inhibitor and chaperone inductor, was able to protect α-Syn-expressing neurons in *Drosophila* (104). Nicotinamide, the principal form of niacin (vitamin B3), has been demonstrated to improve the motor dysfunction in α-Syn transgenic flies through improvement of oxidative mitochondrial dysfunction (105). The dopamine agonists pergolide, bromocriptine, and 2,3,4,5-tetrahydro-7,8-dihydroxy-1-phenyl-1H-3-benzazepine (SK&F 38393), D-519, and D-520 were substantially effective on improvement of locomotor function of α-Syn flies (106, 107). Atropine, the prototypical muscarinic cholinergic receptor antagonist, was effective (106). A potent dopamine D_2_/D_3_ receptor agonist D-607 exhibited significant neuroprotection in a *Drosophila* model of synucleinopathy (108). In addition, HDAC inhibitors such as sodium butyrate or SAHA, and SIRT2 inhibitors have been identified to protect against α-Syn-induced neurotoxicity in flies (109, 110). Taken together, these studies suggest that protein quality control systems, oxidative stress, mitochondrial function, and DA biosynthesis pathways are potential targets for developing therapeutic agents for α-Syn toxicity.

## Modeling GBA-Associated PD in *Drosophila*

Heterozygous mutations in glucocerebrosidase (GCase, encoded by *GBA1* gene) are recently emerging to be the most common known genetic risk factor for PD (111). GCase is a lysosomal protein and homozygous mutations cause Gaucher’s disease, a lysosomal storage disorder (112). As a lysosomal enzyme, GCase is synthesized in the endoplasmic reticulum (ER). At ER, it undergoes N-linked glycosylation on four asparagines. After correctly folded, it processes to the Golgi for further modifications on its N-linked glycans, and finally it traffics to the lysosomes (113). GCase cleaves the β-glucosyl linkage of glucosylceramide (GlcCer) and glucosylsphingosine (GlcSph). Mutations in *GBA* cause accumulation of lipid substrates of GCase such as GlcCer and GlcSph (114). Recent reports demonstrated that mutations in GBA not only contribute to the occurrence of PD but also lead to more significant and rapid cognitive decline in PD (115). Most disease causing mutations of GBA are thought to be dominant-negative mutations that lead to the GBA deficiency, compromised GlcCer metabolism and the subsequent failure of lysosomal mediated degradation of GBA substrates and α-Syn. A severe heterozygous mutation L444P and a mild heterozygous mutation N370S are the most common mutations of GBA in PD. Mutations L444P and N370S cause ER stress, decreased lysosomal GCase, and accumulation of α-Syn aggregates (116, 117). *Drosophila* as one of the major model systems so far for studying GBA-associated PD has revealed significant insights (Table 3).

**Table 3:** *Drosophila* models of glucocerebrosidase (GBA)-associated Parkinson’s disease.

Genetic manipulations	References	GBA variants	Neurodegeneration	Motor activities/life span	Cellular functions
Knockout/knockdown	(118)	dGBA1a^−/−^	ND	Locomotor activity: no changes Life span ↑	ND

		dGBA1b^−/−^ dGBA1a,b^−/−^	Tyrosine hydroxylase (TH) neurons ↓	Locomotor activity ↓↓ Life span ↓↓	Changes in lipid metabolism, accumulation of substrate GlcCer, deficits in lysosomal–autophagy pathway, and abnormality of mitochondria

	(119)	dGBA1a,b^−/−^(ΔTT)	TH neurons: no changes Other neurodegeneration	Locomotor activity ↓ Life span ↓ Memory deficits	Ubiquitinated proteins ↑ α-Syn aggregates ↑ α-Syn expression does not enhance the phenotypes

	(120, 121)	dGBA1a,b^−/−^	TH neurons ↓	Life span ↓	GCase activity ↓

	(122)	dGBA1a-RNAi	TH neurons ↓ Retinal degeneration	Locomotor activity ↓	Proteinase K-resistant α-Syn accumulation when crossed with α-Syn flies

Transgenic	(120, 121)	hGBA-WT	No changes	No changes	No changes

		hN370S hL444P	TH level ↓	Locomotor activity ↓ Life span ↓	GCase activity ↓ ER stress ↑

	(123)	hGBA-WT	ND	ND	Neurodevelopment in fly eyes: no changesER stress: no changes
		hR120W			Neurodevelopment in fly eyes: no changesER stress ↓
		HRecNcil (L444P + A456V + V460V)			Neurodevelopmental defects in fly eyes ↑ER stress ↓

	(124)	hGBA-WT	TH neurons: no changes	Locomotor activity: no changes	ER stress ↑

		hN370S hL444P	TH neurons ↓	Locomotor activity ↓	GCase activity ↓ compared with WT ER stress ↑

*ND, not determined; ↑, increased; ↓, decreased*.

### GBA *Drosophila* Models

#### GBA Knockout/Knockdown *Drosophila* Models

*Drosophila* has two homologs of the human *GBA1* gene, CG31148 and CG31414, which are referred to *dGBA1a* and *dGBA1b*, respectively, and shares 32% amino acid identity. These two genes are found on the same chromosome with the *CG31413* gene in between and show differential tissue expression. *dGBA1b* is expressed in the adult brain at low levels as well as in the adult fat body, whereas *dGBA1a* is predominantly expressed in the adult fly gut but not in the adult brain (FlyAtlas) (125). As GBA mutations in PD show a dominant-negative function, the loss-of-function of GBA was investigated in *Drosophila* by either knockdown or knockout dGBA1.

Kinghorn et al. generated dGBA1a, dGBA1b single knockouts (dGBA1a^−/−^, dGBA1b^−/−^) or dGBA1a/b double knockouts (dGBA1a,b^−/−^) using ends-out homologous recombination (118). dGBA1b^−/−^ and dGBA1a,b^−/−^ showed a significantly decreased survival and age-dependent locomotor deficits compared with control flies while dGBA1a^−/−^ showed opposite phenotype with increased survival and without significant effect on climbing ability over time. As *dGBA1b* is expressed in the adult brain, the study was focusing on dGBA1b^−/−^ and dGBA1a,b^−/−^, both of which showed similar phenotypes (118). Knockout of dGBA resulted in changes in lipid metabolism, accumulation of substrate GlcCer, deficits in lysosomal–autophagy pathway, neurodegeneration, and abnormality of mitochondria. Importantly, mTOR inhibitor rapamycin partially ameliorated the lifespan, locomotor, and starvation phenotypes in dGBA deficient flies (118). Another group used publicly available transposon insertions in dGBA1a and dGBA1b to create deletion of dGBA1 (GBA1Δ^TT^) (119). Using this approach, they removed the majority (first 433aa) of dGBA1b, small potion (33 codons) of c-terminal of dGBA1a, and the whole *CG31413* gene in between. Consistent with the study by Kinghorn et al. GBA1Δ^TT^ homozygotes exhibit shortened lifespan, behavioral phenotypes, memory deficits and neurodegeneration but no DA neuronal loss (119). GBA1Δ^TT^ homozygotes increased accumulation of ubiquitinated proteins and α-Syn aggregates. However, α-Syn expression does not enhance GBA1Δ^TT^ fly phenotypes (119). In addition to these two knockout dGBA1 fly lines, Maor et al. took advantage of two other fly lines, each of which has a minos insertion in dGBA1a and dGBA1b, respectively, to cause premature termination of dGBA1a and dGBA1b. By crossing these two lines, double heterozygous flies have been generated. This fly model exhibited about 30% decrease in GCase activity and has decreased TH immunoreactivity, shortened lifespan, and an age-dependent DA neurodegeneration (120, 121). Besides the knockouts of dGBA1, Suzuki et al. used transgenic RNAi flies to knock down dGBA1a and dGBA1b (122). dGBA1a-RNAi flies exhibited a bout 80–90% decrease in GCase activity while dGBA1b-RNAi flies only showed about 20% decrease. Thus, the study focused on dGBA1a-RNAi flies, which exacerbated the locomotor dysfunction, loss of DA neurons, retinal degeneration, and accumulation of proteinase K-resistant α-Syn in α-Syn-expressing flies (122).

Both knockdown and knockout of dGBA1 in fly have been consistently shown shortened lifespan, behavioral phenotypes and accumulation of α-Syn aggregates, despite of different phenotypes in DA neurodegeneration. These GBA fly models provide useful platforms for further study of GBA function in PD.

#### GBA Transgenic *Drosophila* Models

Heterozygous mutations L444P and N370S are the most common and thought to be dominant-negative mutations of GBA in PD. To investigate GBA functions in PD, transgenic *Drosophila* expressing human WT, N370S and L444P were generated (120, 121, 124). N370S and L444P transgenic flies exhibited significant decreased GCase activity by 82 and 75%, respectively, compared with GBA WT transgenic flies despite equivalent expression levels of GBA protein (124). N370S and L444P transgenic flies consistently showed shortened life span, a progressive climbing defect, increased level of ER stress and DA neurodegeneration (121, 123, 124). This suggests that those transgenic flies can recapitulate some PD signs.

### GBA *Drosophila* Models Reveal GBA Functions in PD

Two major functions of GBA have been implicated in GBA *Drosophila* models. One is the function in ER stress and unfolded protein response (UPR) in the ER. Mutant GCase are recognized as misfolded proteins and undergo various degrees of ER associated degradation. The accumulation of midfolded molecules in the ER activate signaling events known as UPR (120). Immunostaining in GBA transgenic flies revealed that a significant amount of GCase colocalized with ER and N370S and L444P caused abnormal aggregates and swelling within the ER (124). To measure UPR activation, an ER stress reporter transgene Xbp1 was used. N370S and L444P GBA mutations induced significant higher Xbp1 level compared to WT GBA flies, suggesting of an increased level of ER stress (120, 124). Another function revealed by GBA fly models is in lysosomal-autophagic pathway. Using LysoTracker and LC3 as markers to monitor lysosomal and autophagic pathology, Kinghorn et al. demonstrated that enlarged and abnormal lysosomes and accumulated Atg8, the fly LC3 homolog, were present in dGBA knockout fly brains (118). The probable downstream effects of lysosomal-autophagic dysfunction could be the accumulation of p62, a marker for lysosomal-autophagic degradation, and polyubiquitinated proteins (118). While these studies provide significant phenotyping investigation on GBA functions, the detailed molecular mechanisms are still largely unknown. Using these GBA *Drosophila* models, further dissection of the molecules involved in these pathways is warranted.

### GBA *Drosophila* Models as Platforms to Identify Potential Therapeutic Compounds

The clear evidence showed that mutant GCase causes increased ER stress and activated UPR in fly, therefore removal of mutant misfolded GCase by pharmacological chaperones from the ER should at least partially rescue the phenotype. Two chaperones, ambroxol and isofagomine, were previously used to increase amount and lysosomal activity of mutant GCase (126–129). Indeed, ambroxol and isofagomine reduced ER stress, and reversed locomotor deficits in GBA mutant flies *in vivo* (121, 124). This suggests that removal of mutant misfolded GCase from the ER may alleviate PD symptoms. Small chaperones can cross the blood–brain barrier, bind to GCase and stabilize proper folding and ensure delivery to lysosomes. Thus, small chaperones may be applicable for GBA-associated PD.

## Modeling VPS35-Associated PD in *Drosophila*

Mutations in the *VPS35* gene encoding a core subunit of a heteropentameric complex referred to the retromer have recently emerged as a new cause of late-onset, autosomal dominant familial PD (7, 8). The mutation, D620N, has so far been unambiguously identified to cause PD. The VPS35 protein functions as a core component of the retromer, a protein complex that associates with the endosome to facilitate recycling of transmembrane protein cargoes from both endosome-to-Golgi and endosome-to-plasma membrane transport (130). The retromer is a highly conserved multi-protein complex, the core of which consists of the subunits VPS35, VPS29, and VPS26. The only identifiable VPS35 homolog in *Drosophila* is encoded by CG5625.

### VPS35 *Drosophila* Models

#### VPS35 Knockout/Knockdown *Drosophila* Models

Two lines of null mutation in *VPS35* were generated by either imprecise excision of a P-element inserted at the 5′ end of CG5625 (P[EPgy2]CG5625EY^14200^), or by a deletion of nearly 2 kb, which removes the first three exons including the translation start site (*VPS35^MH20^*) (Table 4). Both mutants die at late larval or pupal stages, indicating the essential function of VPS35. VPS35-null mutants and RNAi lines (the Vienna *Drosophila* RNAi Center) were consistently demonstrated to reduce Wingless secretion but not Hedgehog signaling by reducing the recycle of Wntless from endosomes to the trans-Golgi network (131–133). Loss of VPS35 inhibits scavenger receptor ligand endocytosis, causes signaling defects at the NMJ, and leads to over proliferation of blood cells in larvae, which suggests VPS35 has tumor suppressor properties (134). Mechanistically, the endocytic and signaling defects of VPS35 mutants maybe due to VPS35 negatively regulation of actin polymerization (134). As these studies were at early stages before mutations of VPS35 has been identified to associate with PD, the PD pathological phenotypes in VPS35 knockout or knockdown mutants were not investigated. In recent studies, knockdown of VPS35 in *Drosophila* induced the accumulation of the detergent-insoluble α-Syn in the brain and exacerbated locomotor deficits, compound eye disorganization, and interommatidial bristle loss in α-Syn transgenic flies (135). These findings indicate that the retromer may play a crucial role in α-Syn degradation. The loss of *Drosophila* VPS35 (dVPS35) affects synaptic vesicle recycling, DA synaptic release and sleep behavior (46). The manipulation of *Drosophila* LRRK2 dLRRK together with Rab5 and Rab11 improves the VPS35 synaptic phenotypes (46). Taken together, VPS35 knockout/knockdown *Drosophila* models mimic some pathogenesis of PD, indicating that these fly models could be useful platforms to study VPS35-associated PD.

**Table 4:** *Drosophila* models of vacuolar protein sorting 35 (VPS35)-associated Parkinson’s disease.

Genetic manipulations	References	VPS35 variants	Neurodegeneration	Motor activities/life span	Cellular functions
Knockdown	(135)	dVPS35 siRNA in α-Syn transgenic fly	ND	Locomotor activity ↓	Accumulation of the detergent-insoluble α-Syn, cathepsin D activity ↓ Mild eye disorganization

Knockout	(46)	dVPS35^−/−^	Tyrosine hydroxylase (TH) neurons: no changes	ND	Defects on synaptic vesicle recycling, dopaminergic synaptic release and sleep behavior associated with dopaminergic activity; genetic interaction with leucine-rich repeat kinase 2 and Rab5, Rab

	(136)	dVPS35^−/−^	ND	Locomotor activity ↓	Synaptic overgrowth ↓

Transgenic	(137)	hVPS35-WT hL774M	TH neurons: no changes	No changes	Sensitive to rotenone: no changes

		hD620N hP316S	TH neurons ↓	Locomotor activity ↓ Life span ↓	Sensitive to rotenone ↑

	(136)	dVPS35-WT dD650N dR550W dL800M hVPS35-WT hD620N hR524W hL774M	No retinal degeneration	Locomotor activity: no changes Life span: no changes	D620N mutation confers a partial loss of function; VPS35 genetically interacts with Parkin

*ND, not determined; ↑, increased; ↓, decreased*.

#### VPS35 Transgenic *Drosophila* Models

Vacuolar protein sorting 35-linked PD is inherited as a dominant trait, which may imply that the mutation of VPS35 has a gain-of-function toxicity (Table 4). One study demonstrated that VPS35 D620N transgenic flies led to late-onset loss of DA neurons, locomotor deficits, shortened lifespans, and increased sensitivity to a PD-linked environmental toxin, rotenone (137). However, Malik and colleagues did not find evidence of dominant toxicity from any variants including the pathogenic D620N mutation, even with aging. By a definitive test to determine whether transgene expression can rescue endogenous VPS35 mutant phenotypes, they concluded that the D620N mutation confers a partial loss of function (136). This notion is further supported by other studies in fly or mouse systems that VPS35 DN mutation acts as a dominant-negative function (47, 48, 138, 139).

### VPS35 *Drosophila* Models Reveal VPS35 Functions in PD

To date, VPS35 *Drosophila* models have revealed three major functions of VPS35 in trafficking pathways in neuronal system. First, VPS35 regulates synaptic vesicle endocytosis through the endosomal pathway. Loss of VPS35 increases the number of synaptic boutons of the NMJ in larval motor neurons (46, 134). It has been demonstrated that VPS35 cooperates with LRRK2 to regulate synaptogenesis, synaptic dynamics and endocytosis, and synaptic vesicles regeneration through the Rab-mediated endocytic pathway (46). Importantly, it in turn regulates DA activity and survival, a key element of PD etiology (46). Second, VPS35 mediates endolysosomal and Golgi apparatus sorting. Wild-type VPS35, but not a familial PD-associated mutant form, can rescue LRRK2 led to endolysosomal and Golgi apparatus sorting defects (48). In addition, it has been reported by several groups that VPS35 functions in endosome-to-Golgi retrieval are required for Wingless secretion (131–133). However, whether this function is related to DA neurodegeneration is unknown. Third, VPS35 functions in lysosomal degradation pathway. VPS35 dysfunction impairs the maturation of a lysosome protease cathepsin D in regulating the proteolytic pathway that is important for α-Syn metabolism, and in turn exacerbates neurotoxicity and causes eye degeneration and motor disability (135). These findings indicate that VPS35 may play a crucial role in α-Syn degradation and might thereby contribute to the pathogenesis of the disease. While it remains unclear if these functions are causally for DA neurodegeneration caused by VPS35 PD mutant, these studies have provided important insights into cellular pathways that are perturbed by VPS35 mutations in neurons.

## Convergent Mechanisms

Dissecting genetic interaction among PD genes will be crucial to establish convergent functional pathways of these genes or risk factors. *Drosophila* as a classic genetic model provides powerful tools to study genetic interactions between different genes. Genetic dissection revealed that LRRK2 interacts with other PD genes or risk factors such as Parkin, DJ-1, PINK1, VPS35, and RAB7L (31, 46–48) and implicated several potential functions. Genetic interaction between LRRK2 and VPS35 or Rab7L indicates LRRK2 function in retromer and lysosomal pathways (46–48). Genetic interaction between LRRK2 and Parkin or PINK1 indicates LRRK2 function in mitochondria dysfunction and also suggests that dominant PD genes may act *via* common pathways with the recessive PD genes (31). Furthermore, VPS35 genetically interacts with *Parkin* but interestingly not with *PINK1*(136). Notably, α-Syn, LRRK2, and PINK1 have recently been linked to Rab proteins (48, 92–98), and the manipulation of *Drosophila* LRRK2 dLRRK together with Rab5 and Rab11 improves the VPS35 synaptic phenotypes (46). All the studies are convergent to implicate an important emerging role for defects in trafficking pathways. The accumulation of altered proteins including α-Syn and damaged mitochondria ultimately might overwhelm the disposal mechanisms, in turn cause DA neurodegeneration.

## Concluding Remarks

While the rodent models generally attack more attention and efforts on studying human disorders because of their high conservation of basal ganglia circuit with human, modeling PD in rodents using genetics has been viewed as difficult (23, 140). The rodent models of PD could not fulfill all the key features of PD (140). The reason that the rodents are “imperfect” for modeling PD might be compensatory mechanisms in the rodents, and/or incomplete penetrance of some PD gene mutations such as LRRK2 disease causing mutations in human, and/or the combination effects of non-cell-autonomous and cell-autonomous processes (23, 140).

To this caveat, *Drosophila* models have provided significant contributions to our understanding of the mechanisms of PD pathogenesis in a comparatively short time frame and cost effective mode. Overexpression of PD dominant traits (LRRK2 and α-Syn) or knockout of dominant-negative genes (GBA and VPS35) in fly has been consistently demonstrated to mimic the essential PD signs such as DA neurodegeneration and behavioral deficits. Based on these fly models, genetic modifiers and small molecular compounds have been rapidly identified. Moreover, the combination roles of the genetic and environmental factors such as oxidative stress have been explored in PD. The important functions of LRRK2 in trafficking and protein translation, the critical contribution of α-Syn aggregation and phosphorylation, were initially discovered in fly. The *Drosophila* models so far are one of the major model systems to study GBA function in PD. Thus, the use of *Drosophila* models opened tremendous opportunities to explore the basic function of disease causing genes and to model the disease pathogenesis.

However, *Drosophila* is a relatively simple model organism, far less complex brain circuit than humans. For example, *Drosophila* does not have α-Syn homolog and a true human LRRK2 homolog. α-Syn neuropathology in the form of LBs is the hallmark of PD pathogenesis. Whether α-Syn is required for developing PD models has been raised. In addition, *Drosophila* has limited cell death effectors. Some aspects of human diseases may not be evident in fly. Thus, validation of findings from *Drosophila* to mammalian systems, including rodent models, human postmortem tissue, and human DA neuronal cultures, is warranted. Therefore, the strategy would be to identify the basic aspects of the underlying disease mechanisms using simple organism model systems such as *Drosophila* and further characterize and validate the findings in mammalian conditions.

## Author Contributions

YX and JY analyzed the literatures and wrote the manuscript.

## Conflict of Interest Statement

The authors declare that the research was conducted in the absence of any commercial or financial relationships that could be construed as a potential conflict of interest.

## Abbreviations

PD, Parkinson’s disease; LRRK2, leucine-rich repeat kinase 2; PINK1, phosphatase and tensin homolog deleted on chromosome 10-induced putative kinase 1; VPS35, vacuolar protein sorting 35; GBA, glucocerebrosidase; DA, dopaminergic; SNpc, substantia nigra pars compacta; EMS, ethyl methanesulfonate; SJ1, synaptojanin 1; EndoA, endophilin A; GSK3β, glycogen synthase kinase 3β; GWASs, genome-wide association studies; LBs, Lewy bodies; α-Syn, α-synuclein; UPS, ubiquitin-protease system; HDAC6, histone deacetylase 6; TRAP1, tumor necrosis factor receptor associated protein-1; ER, endoplasmic reticulum; GlcSph, glucosylceramide (GlcCer) and glucosylsphingosine; GD, Gaucher’s disease; UPR, unfolded protein response.
